# A digital decision support system (selfBACK) for improved self-management of low back pain: a pilot study with 6-week follow-up

**DOI:** 10.1186/s40814-020-00604-2

**Published:** 2020-05-23

**Authors:** Louise Fleng Sandal, Cecilie K. Øverås, Anne Lovise Nordstoga, Karen Wood, Kerstin Bach, Jan Hartvigsen, Karen Søgaard, Paul Jarle Mork

**Affiliations:** 1 Department of Sport Science and Clinical Biomechanics, University of Southern Denmark (UoSD), Odense, Denmark; 2 Department of Public Health and Nursing, Norwegian University of Science and Technology (NTNU), Trondheim, Norway; 3 Nordic Institute of Chiropractic and Clinical Biomechanics, Odense, Denmark; 4 Department of Computer Science, Norwegian University of Science and Technology (NTNU), Trondheim, Norway; 5 School of Medicine, Dentistry & Nursing, University of Glasgow, Glasgow, UK

**Keywords:** Low back pain, Self-management, App, Case-based reasoning, mHealth, Recommender system, Artificial intelligence

## Abstract

**Background:**

Very few of the publicly available apps directed towards self-management of low back pain (LBP) have been rigorously tested and their theoretical underpinnings seldom described. The selfBACK app was developed in collaboration with end-users and clinicians and its content is supported by best evidence on self-management of LBP. The objectives of this pilot study were to investigate the basis for recruitment and screening procedures for the subsequent randomized controlled trial (RCT), to test the inclusion process in relation to questionnaires and app installation, and finally to investigate the change in primary outcome over time.

**Methods:**

This single-armed pilot study enrolled 51 participants who had sought help for LBP of any duration from primary care (physiotherapy, chiropractic, or general practice) within the past 8 weeks. Participants were screened for eligibility using the PROMIS-Physical-Function-4a questionnaire. Participants were asked to use the selfBACK app for 6 weeks. The app provided weekly tailored self-management plans targeting physical activity, strength and flexibility exercises, and education. The construction of the self-management plans was achieved using case-based reasoning (CBR) methodology to capture and reuse information from previous successful cases. Participants completed the primary outcome pain-related disability (Roland-Morris Disability Questionnaire [RMDQ]) at baseline and 6-week follow-up along with a range of secondary outcomes. Metrics of app use were collected throughout the intervention period.

**Results:**

Follow-up data at 6 weeks was obtained for 43 participants. The recruitment procedures were feasible, and the number needed to screen was acceptable (i.e., 1.6:1). The screening questionnaire was altered during the pilot study. The inclusion process, answering questionnaires and app installation, were feasible. The primary outcome (RMDQ) improved from 8.6 (SD 5.1) at baseline to 5.9 (SD 4.0) at 6-week follow-up (change score 1.8, 95% CI 0.7 to 2.9). Participants spent on average 134 min (range 0–889 min) using the app during the 6-week period.

**Conclusion:**

The recruitment, screening, and inclusion procedures were feasible for the subsequent RCT with a small adjustment. The improvement on the RMDQ from baseline to follow-up was small. Time pattern of app usage varied considerably between the participants.

**Trial registration:**

NCT03697759. Registered on August 10, 2018. https://clinicaltrials.gov/ct2/show/NCT03697759

## Background

Self-managing disease, such as chronic illness like low back pain (LBP), is positively affected by receiving social support [1, 2]. Using mHealth applications (apps) to support and reinforce desired self-management behavior has been put forward as a promising way to increase the effectiveness of self-management interventions [3–5]. Clinical guidelines for LBP commonly recommend self-management [6–8]. Self-management may include several different components, such as self-monitoring of symptoms, physical activity, regular strength/flexibility exercises, and patient education.

Within recent years, the number of mHealth apps has grown substantially [9, 10]. The number of available mHealth apps increased from 153.403 in 2015 to 318.572 in 2017 [10]. Of the available mHealth apps, 60% concerned wellness management, 40% targeted health condition management, with 16% of all mHealth apps being disease specific [10]. Most commonly, mental health and behavioral disorders, diabetes, hypertension, and heart and circulatory conditions were targeted [10–12] and only 7% of the disease specific apps targeted musculoskeletal conditions [10]. Despite the worldwide prevalence and impact, apps concerning musculoskeletal disorders are underrepresented. Thus, there is a need for developing evidence-based mHealth apps that support management of musculoskeletal disorders such as LBP. Moreover, such apps should be rigorously tested to document their effectiveness. This will be important for informing end-users and health care professionals about the expected benefit of using such tools.

Very few of the available mHealth apps have been rigorously tested and their theoretical underpinning is most often poorly documented [13, 14]. For example, a recent systematic review identified 61 apps for self-management of LBP and concluded that none of the apps were tested in a randomized controlled trial (RCT) and that the app content in general had low quality [14]. An increase in the number of clinical trials investigating safety and effectiveness of mHealth apps is however evident as 135 trials were registered to utilize medical apps at the trial registry www.clinicaltrials.gov in 2013, increasing to 300 in 2015 [15] and 869 in 2017 [10].

In the selfBACK project, a decision support system (DSS) has been developed to support self-management of LBP [16, 17]. The decision support incorporates evidence-based and tailored recommendations for self-management delivered via an app. Where RCTs aim to evaluate the effectiveness of a given intervention, pilot trials often aim to assess the feasibility of the intervention as well as testing recruitment basis and procedures for the following RCT [18]. The objectives of this pilot study were multiple: to investigate the basis for recruitment and screening procedures for the subsequent RCT, to test the inclusion process in relation to completing questionnaires online and installation of apps, and to investigate the change in primary outcome over time.

## Methods

### Design

This single-armed pilot study was performed in advance of an RCT. All participants were fully informed of the purpose and allocated to the selfBACK intervention. The study followed an a priori defined protocol that was registered before the recruitment of participants was initiated (NCT03697759). The study is reported in accordance with the CONSORT guideline [18]. Approval for the pilot trial was obtained from the regional Ethics committees in Denmark (S-20182000-24) and Norway (2018/791). Approval from the data protection agency was obtained for Denmark (18/17955) through the University of Southern Denmark. In Norway, this was covered by the Ethics committee approval. This pilot study was a part of the larger selfBACK project funded by the European Union Horizon 2020 research and innovation program (grant agreement no 689043).

### Participants

Inclusion and exclusion criteria are described in Table 1.

**Table 1:** Inclusion and exclusion criteria

Inclusion criteria	
• Danish or Norwegian adults over 18 years of age • LBP of any duration, who have sought care for their LBP within the past 8 weeks from primary care (primary care defined as general practice, physiotherapy, chiropractic serving, or a specialized outpatient hospital facility [Denmark]) • Mild-to-severe disability due to LBP • Own and regularly use a smartphone with internet access • Have a working email address and access to a computer with internet access	
Exclusion criteria	
• Unable to speak, read or understand the national language (Danish/ Norwegian) • Cognitive impairments or learning disabilities limiting participation • Serious mental illness • Physical illnesses or conditions limiting participation • Terminal illness • Inability to take part in exercise/physical activity • Fibromyalgia diagnosed by a health care professional • Pregnancy • Previous back surgery	

*LBP* low back pain

### Recruitment procedure

Recruitment of participants took place in Trondheim, Norway, and Odense, Denmark, from August to November 2019. The flow of the participants throughout the study is shown in Fig. 2 and described in detail elsewhere [19]. People with LBP could be referred to the pilot study after seeing a primary care clinician (i.e., general practitioner, physiotherapist, chiropractor). Additionally, in Denmark, recruitment was also conducted from the Spine Center of Southern Denmark that performs diagnostic assessment of people with back-related problems. Participants identified as potentially eligible by the clinician were given verbal and/or written information about the pilot study by the referring clinician and asked to contact the research team by email or phone or provide their contact information on a sign-up sheet allowing the research team to contact them. Recruitment rates from the different types of primary care providers and the Spine Center were tracked. In addition, experiences concerning the type of communication with clinics and clinicians that were most successful and how to maintain a good contact with the recruiting clinics were discussed during weekly meetings in the research teams, summarized, and considered when planning for the RCT.

### Screening procedure

Interested participants were contacted via phone by the research team and screened for eligibility using a standardized screening form with the inclusion and exclusion criteria (Additional file 2). The researchers read the questions aloud from the screening form to participants and recorded their answers. If eligible and willing to participate, the participants gave their verbal consent to participate, received a link to complete a baseline questionnaire, and were invited to a meeting with the researcher. At this meeting, participants received information on the study and gave their written informed consent to participate. The inclusion criterion for mild-to-severe disability was assessed using the PROMIS-Physical Functioning 4 questionnaire (PROMIS-PF4). This is an outcome aiming at measuring disability, which is a recommended core outcome in clinical trials for LBP populations [20]. The questionnaire consists of 4 items asking people with LBP to rate their difficulty performing four activities (chores, stair walking, 15 min walk, running errands) on a 5-point Likert scale ranging from “without any difficulty” to “unable to perform.” Total scores range between 4 and 20 with lower scores indicating more disability. A score of 16 or below was indicative for eligibility.

### Intervention

**Fig. 1:**
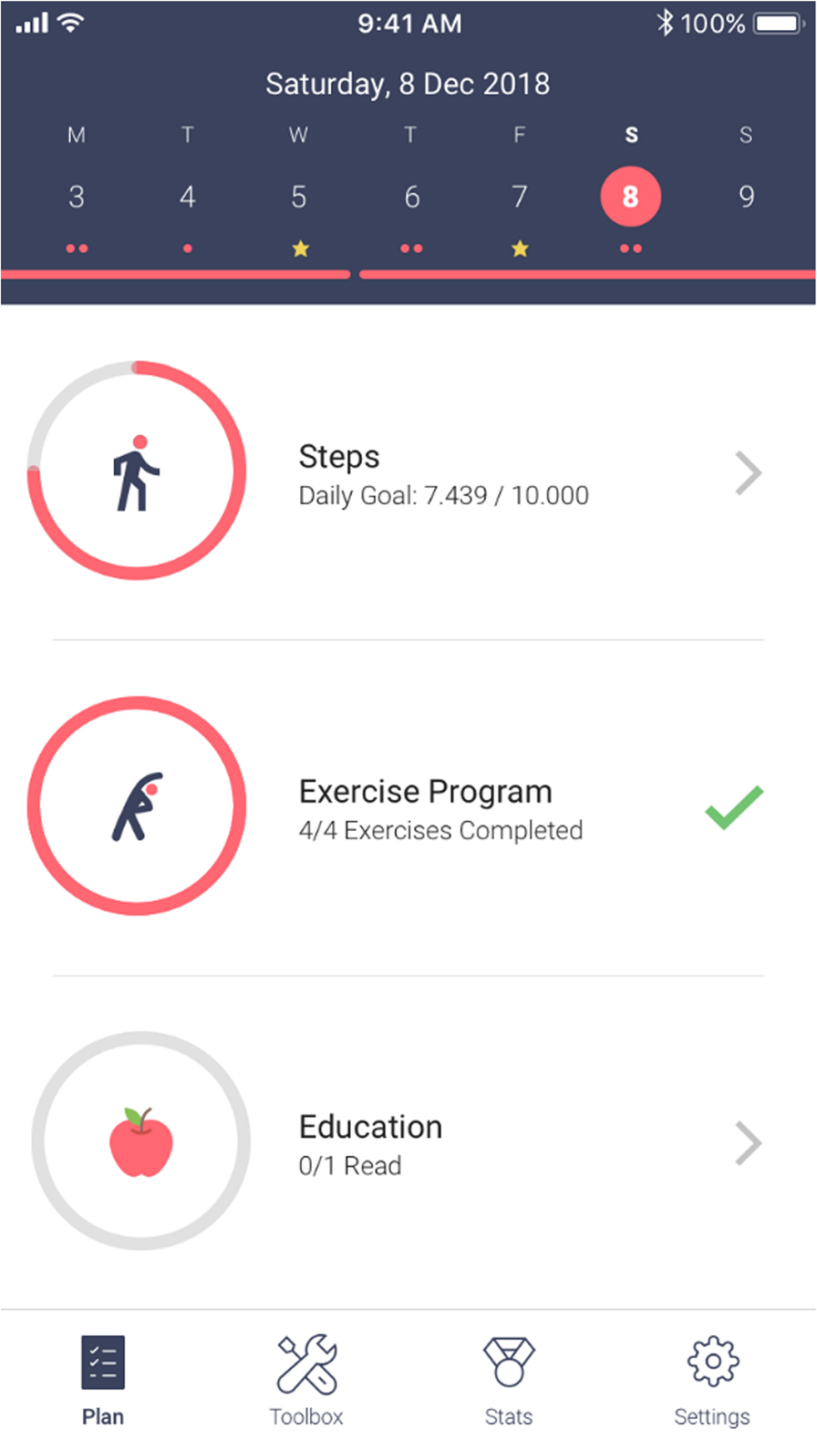
Screenshot of the selfBACK app plan screen showing the three main components of a weekly self-management plan

All participants had the selfBACK app installed on their smartphone during a visit with the research team who helped with the app installation and answered any questions that participants might have. Hereafter, participants were asked to use the selfBACK app for 6 weeks to self-manage their LBP in addition to receiving their usual care. The selfBACK app is an add-on to and not a substitution for treatment or seeking advice from HCPs. The development of the selfBACK app, its underlying structure, and development of content have been described in detail elsewhere [16]. In brief, the selfBACK app provides weekly tailored self-management plans targeting three main activities: (1) general physical activity (i.e., number of steps) measured by a step-detecting wristband, (2) strength and flexibility exercises, and (3) patient education (Fig. 1). In addition, the app also provides access to variety of tools and information on management of LBP that the participants could use at their convenience. Examples of tools are a goal-setting tool, mindfulness audios, pain relieving exercises, and general information about the nature of LBP. The feasibility of the app was assessed in a mixed-methods study prior to this pilot study. The results will be reported in a separate publication.

### Demographics and self-reported outcomes

Demographics and self-reported outcomes were collected using a web-based questionnaire. Demographics included gender, age, height/weight (to calculate body mass index [BMI]), civil status and children, education, employment status, and work characteristics. All outcomes were collected at baseline and after 6 weeks (Table 2) using a web-based questionnaire to gauge its feasibility. The self-reported outcomes included the recommended core set of outcomes for LBP trials [20, 38–40] (physical functioning, pain intensity, and health-related quality of life). Only results from the primary outcome (Roland-Morris Disability Questionnaire [RMDQ]) will be reported on here, all other scores are given in Additional file 1.

**Table 2:** Self-reported outcomes assessed at baseline and 6-week follow-up

Domain	Measure	Description
Pain-related disability	RMDQ [21–23]	24 items on ability to perform everyday tasks, range 0–24, higher scores indicate higher pain-related disability
LBP intensity, average past week	NRS [24]	LBP intensity rated on an NRS, range 0–10, higher scores indicate higher LBP intensity
Fear-avoidance	FABQ [25, 26]	5 items on LBP and physical activity, range 0–24, higher scores indicate higher fear-avoidance beliefs
Self-efficacy	PSEQ [27, 28]	10 items on confidence to cope with LBP, range 0–60, higher scores indicate higher confidence
Work ability	WAI [29]	Single item on work ability rated on a NRS, range 0–10, higher scores indicate better work ability
Physical functioning	PSFS [30, 31]	Participants identify up to 2 important activities and rate the ability to perform these activities, range 0–10, higher scores indicate better functioning
Health-related QoL	EQ-5D [32]	5 items (mobility, self-care, usual activities, pain/discomfort, anxiety/depression) and a 100-mm vertical VAS on health, higher scores indicate better health
Mental health	PHQ-8 [47]	8 items on symptoms of depression, range 0–24, higher scores indicate higher level of depressive symptoms
Perceived stress	PSS [33]	10 items on perception of stress, range 0–40, higher scores indicate higher level of perceived stress
Illness perception	BIPQ [34]	8 items on perception of how LBP interferes with everyday life, range 0–80, higher scores indicate more threatening perception of LBP
No. of pain sites	Pain mannequin	No. of body sites with current pain, range 0–9, including the following body regions: neck, shoulders, upper back, elbows, lower back, wrists/hands, hips/thighs, knees, and ankles/feet.
Activity limitation		2 items (yes/no) on whether LBP has reduced activity during work and/or leisure
Leisure time physical activity	SGPALS [35]	4 categories ranging from sedentary to regular hard physical activity
Sleep		4 items (problems falling asleep, waking up repeatedly, waking up too early, and daytime sleepiness), range 0–12, higher scores indicate more severe sleep problems
Satisfaction	PASS [36]	Single item on whether an acceptable symptom state has been achieved (yes/no). Assessed only at 6-week follow-up
Perceived effect	GPE [37]	Single item on perception of effect from the intervention, scored on balanced scale ranging from “very much worse” to “very much better.” Assessed only at 6-week follow-up

*RMDQ* Roland-Morris Disability Questionnaire, *LBP* low back pain, *NRS* numerical rating scale, *FABQ* fear avoidance-belief questionnaire; *PSEQ* pain self-efficacy questionnaire, *PSFS* Patient Specific Functional Scale, *PSS* Perceived Stress Scale, *QoL* quality of life; *EQ-5D* EuroQoL 5 dimensions, *VAS* visual analogue scale, *BIPQ* brief illness-perception questionnaire, *PHQ*-8 Patient Health Questionnaire, *WAI* work ability index, *SGPALS* Saltin-Grimby Physical Activity Level Scale, *PASS* patient acceptable symptom state, *GPE* global perceived effect

### App usage

Participants’ use of the app was tracked using the Matomo software (https://matomo.org/) that registers participants’ time and interactions in the app. The following variables were extracted to illustrate app usage: number of total visits to the app, number of days the app was visited, number of daily visits to the app, total time spent in the app, number of self-management plans generated, and achievements on physical activity, exercises, and education (i.e., percentage completion of the recommendation for each of these activities).

### Statistical analysis

Descriptive statistics were used to describe the baseline characteristics of the included participants and the change in the primary outcome from baseline to 6-week follow-up. The baseline characteristics were described as frequencies and means ± standard deviation (SD), the self-reported outcomes were described as means ± SD for baseline and 6-week follow-up, whereas change scores are presented as means with 95% confidence intervals (CI). App usage data is presented as group means and range.

No sample size calculations were performed as this pilot study aimed to investigate the recruitment, screening, and inclusion procedures and to investigate the size of change in score for the primary outcome for the subsequent RCT, rather than to investigate effect or statistical significance of this change from the intervention. The expected sample size for the pilot study was consequently based on feasibility. We aimed to include 70–80 participants (45–60 in Denmark and 15–20 in Norway) within a 3-month recruitment period.

## Results

**Fig. 2:**
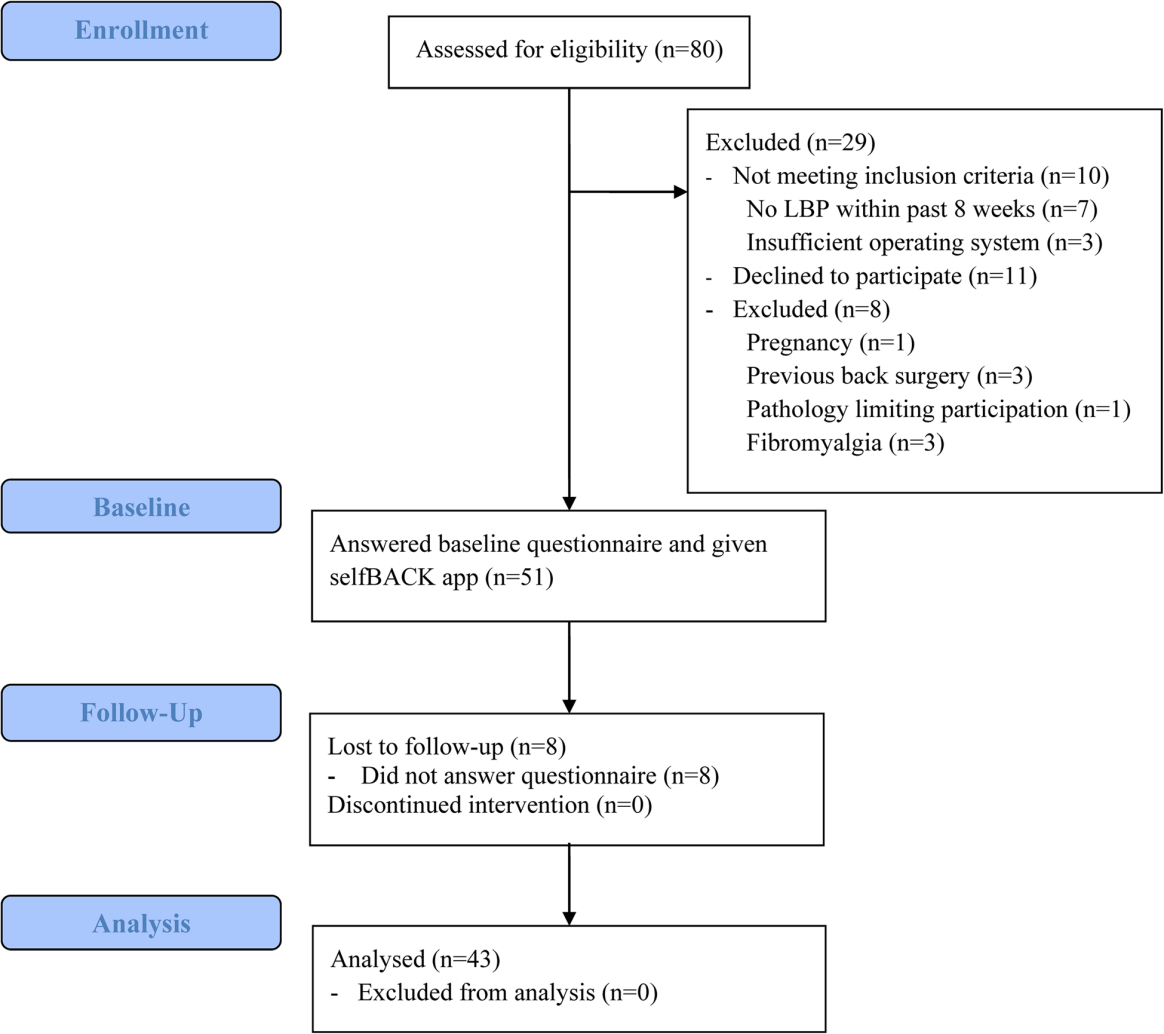
Flow of the participants throughout the study. LBP low back pain

Flow of participants throughout the study is shown in Fig. 2. A total of 93 potential participants consented to be contacted for participation in the pilot study. Of these, we reached 80 potential participants who were assessed for eligibility. After the assessment for eligibility, 51 participants were included.

### Participant characteristics

Characteristics of the study sample are presented in Table 3. Overall, the included participants were middle aged (mean age 45.5 years [SD 15.0 years]), slightly overweight (mean BMI 27.2 kg/m^2^), predominantly female (58%), and living with others (72%). Most participants reported to have had LBP lasting for more than 12 weeks during the current episode (58%) with infrequent use of pain medication (i.e., 55% reported that they never or seldom use pain medication). Furthermore, most of the participants reported to be currently employed in a full-time (59%) or part-time (14%) position.

**Table 3:** Characteristics of the study sample

Variable	
Age (years), mean (SD)	45.5 (15.0)
Body mass index (kg/m^2^), mean (SD)	27.2 (5.5)
Female, no (%)	29 (58%)
Family status	
Living alone, no (%)	14 (28%)
Living with partner, no (%)	17 (33%)
Living with partner/parents and children, no (%)	17 (33%)
Living with children, no (%)	3 (6%)
Education	
10 years, no (%)	1 (2%)
12 years, no (%)	14 (27%)
13 years or more, no (%)	36 (71%)
Employment	
Full-time, no (%)	30 (59%)
Part-time, no (%)	7 (14%)
Full-time housework, no (%)	1 (2%)
Compulsory military service, no (%)	3 (6%)
Retired, no (%)	4 (8%)
Other, no (%)	6 (12%)
Work characteristics* (*n* = 38)	
Sitting, no (%)	19 (50%)
Walking, no (%)	8 (21%)
Walking and lifting, no (%)	10 (26%)
Heavy physical labor, no (%)	1 (3%)
LBP, duration of current episode	
1 week, no (%)	9 (18%)
4 weeks, no (%)	2 (4%)
12 weeks, no (%)	10 (20%)
More than 12 weeks, no (%)	30 (58%)
LBP, frequency within past year	
7 days, no (%)	2 (4%)
30 days, no (%)	9 (18%)
Above 30 days, no (%)	22 (43%)
Every day, no (%)	18 (35%)
Use of pain medication	
Never or seldom, no (%)	28 (55%)
Less than once weekly, no (%)	12 (25%)
Weekly, no (%)	5 (10%)
Daily, no (%)	6 (12%)

*The question about work ability was only asked to participants who reported to be in full-time or part-time work

SD standard deviation, LBP low back pain

### Primary and secondary outcomes

The RMDQ score, which is the primary outcome in the subsequent RCT, improved 1.8 points over the 6-week intervention period (95% CI − 2.9 to − 0.7) (Table 4). Most secondary outcomes improved significantly from baseline to 6-week follow-up, but the numerical differences were modest (Additional file 1).

**Table 4:** Scores for the RMDQ at baseline, 6-week follow-up, and change score

	Baseline (*n* = 51) mean (SD)	6 weeks (*n* = 43) mean (SD)	Change score (*n* = 43) mean (95% CI)
RMDQ (range 0–24)	8.6 (5.1)	5.9 (4.0)	− 1.8 (− 2.9 to − 0.7)

*RMDQ* Roland-Morris Disability Questionnaire, *SD* standard deviation, *CI* confidence interval

### App usage

Table 5 shows the app usage during the 6-week intervention period. This included usage of the app content and responding to the weekly tailoring questions to have a new self-management plan created. On average, participants visited the app 65 times during the study period, spent 134 min in the app, and visited the app on 22 of the 42 possible days during the intervention period. However, inter-individual variation in app use was considerable, e.g., total number of visits ranged from 1 to 188 and time spent in the app ranged from 0 to 889 min.

**Table 5:** App use during the 6-week study period for the 51 participants enrolled in the study

	Mean (range)
Time spent in app (minutes)	134 (0 to 889)
Total no. of visits	65 (1 to 188)
No. of days visiting the app	22 (1 to 47)^*^
No. of visits pr. day on days the app was visited	3 (1 to 5)
No. of self-management plans created	4 (0 to 8)

^*^The maximum possible number of days visiting the app was 47, which is above the 42 days of the intervention. Users were sent the invitation to complete the 6-week follow-up questionnaire after 6 weeks but may be delayed in answering the questionnaire. This results in more days to use the app than the given 6 weeks.

**Fig. 3:**
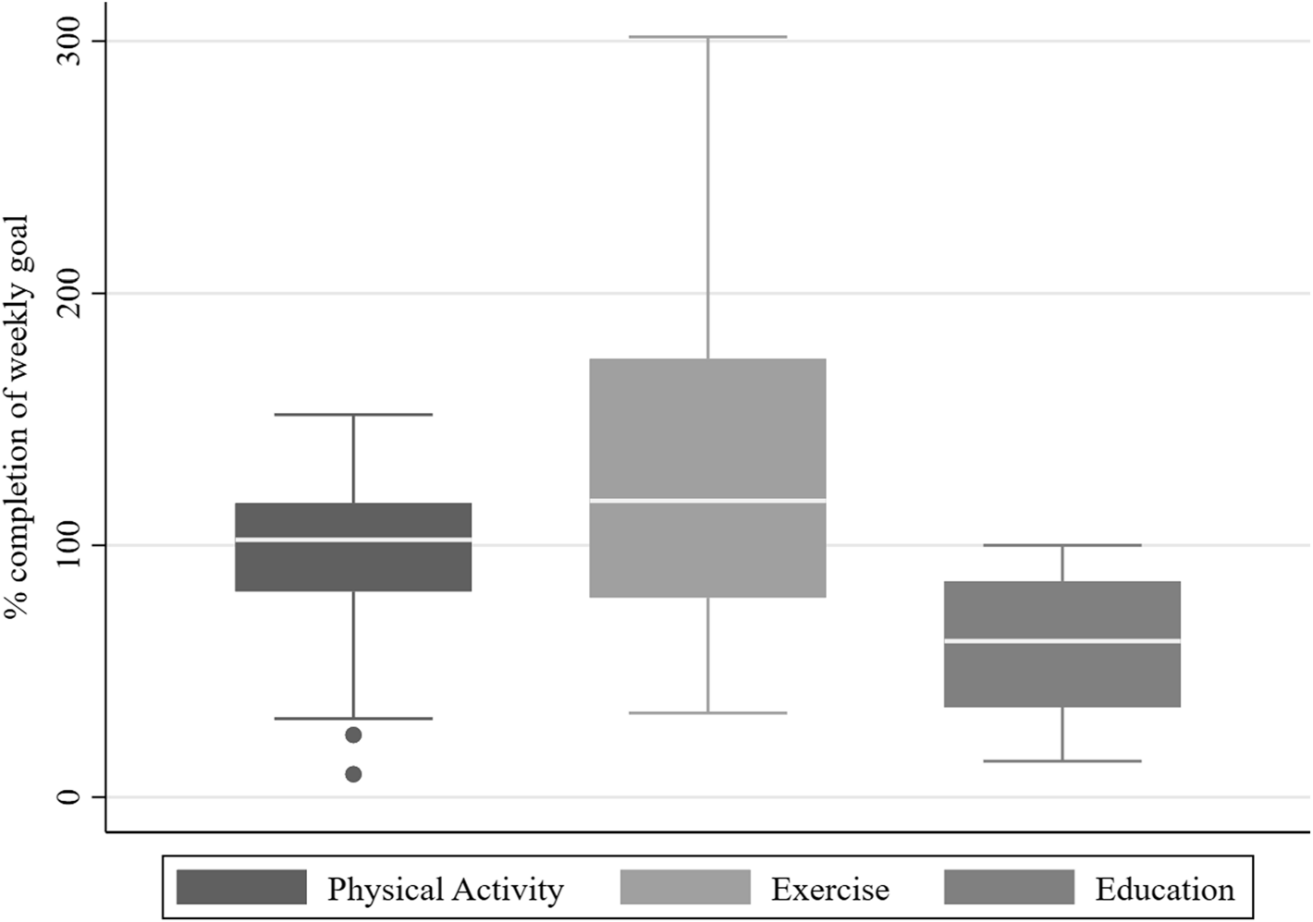
Box-plot showing the completion levels for physical activity (i.e., no. of steps), exercise, and education. Physical activity is shown as percentage completion of the daily step goal (e.g., 130% equals goal achieved plus 30% additional steps). Strength and flexibility exercises are shown as exercise volume (e.g., 100% equals 3 weekly exercise sessions of any duration). Education is shown as the percentage of the suggested educational messages that have been read (e.g., 100% means that all the 7 messages for a week have been read)

Figure 3 shows a box-plot of the average completion levels for the weekly goals in the tailored self-management plans. Physical activity is shown as percentage completion of the daily step goal (e.g., 130% equals goal achieved plus 30% additional steps). Strength and flexibility exercises are shown as exercise volume (e.g., 100% equals 3 weekly exercise sessions of any duration). Education is shown as the percentage of the suggested educational messages that have been read (e.g., 100% means that all the 7 messages for a week have been read). Overall, participants had high completion rates of the weekly goals across all categories: physical activity, exercise, and education.

### Recruitment

Table 6 shows the number of participants screened from the different types of primary care clinics and the number needed to screen to include one patient. Overall, most participants were recruited from physiotherapy and chiropractic clinics. The average number needed to screen was 1.6.

**Table 6:** Recruitment rates and number needed to screen for the different types of clinics

	Physiotherapy clinics	Chiropractic clinics	General practice	Spine Center (DK only)	Total
Norway					
No. screened (%)	12 (41.4)	8 (27.6)	9 (31.0)	–	29
No. included (%)	8 (40.0)	6 (30.0)	6 (30.0)	–	20
No. needed to screen	1.5	1.3	1.5	–	1.5
Denmark					
No. screened (%)	13 (25.5)	26 (51.0)	0 (0.0)	12 (23.5)	51
No. included (%)	12 (38.7)	12 (38.7)	0 (0.0)	7 (22.6)	31
No. needed to screen	1.0	2.2	–	1.7	1.6
Total					
No. screened (%)	25 (31.3)	34 (42.5)	9 (11.3)	12 (15.0)	80^*^
No. included (%)	20 (39.2)	18 (35.3)	6 (11.8)	7 (13.7)	51
No. needed to screen	1.3	1.9	1.5	1.7	1.6

^*^The table provides information on the participants screened. Additionally, 13 participants consented to be contacted but we were unable to reach and screen these (Denmark *n* = 9, Norway *n* = 4)

### Screening

Initially, the inclusion criteria “mild to severe disability due to LBP” was assessed using the PROMIS-PF4 [20, 41–44]. A cutoff point of 16 or below indicated eligibility. This cutoff point was set as a best guess as no studies using the PROMIS-PF4 on a LBP population was available at the time of the pilot study. During the pilot study, we experienced that many of the screened participants seemed to have pain-related disability but did not fall below the cutoff point. We looked at the first 31 participants included, of those, only nine would have been included if enforcing the cutoff point. Consequently, the cutoff point of 16 or below for the PROMIS-PF4 score was not enforced as cutoff point for inclusion to the study for any of the screened LBP patients. We observed a mismatch between the RMDQ score reported at baseline for participants with the same PROMIS-PF4 score. For example, eight participants had a PROMIS-PF4 score of 20 (no disability), but their RMDQ scores ranged between 0 and 18 (no disability to severe disability). We therefore decided to replace the PROMIS-PF4 with the RMDQ in the screening procedure for assessing disability in the remaining part of the pilot study. This substitution allowed us to assess the feasibility of using the RMDQ as the screening tool on the phone for the subsequent RCT. Overall, using the RMDQ for screening proved feasible, i.e., the time used for screening did not increase compared to the PROMIS-PF4 and it was easy for the potential participants to understand and respond to the questions. For the subsequent RCT, a score of ≥ 6 on the RMDQ was used to indicate eligibility. However, this cutoff point was not enforced in the current study and all patients referred with LBP were included if matching the remaining eligibility criteria.

The standardized screening form listed questions related to the inclusion- and exclusion criteria (Additional file 2). Our experiences when screening participants on the phone resulted in a revision of screening questions on terminal illness, cognitive impairments, and serious mental illness as several participants reacted negatively to the wording of these questions. Consequently, the questions concerning these conditions were reworded and combined with the screening question about physical conditions limiting participation in the intervention (e.g., problems getting down on the floor and up again to perform strength and flexibility exercises). This did not result in any change in the characteristics of the target population, but rather consisted of a clarification to ensure that the potential participants understood the requirements for participating in the intervention. On average, it took about 15 min to screen a participant on the phone.

### Data collection and app installation

Sending a link to participants via email to access and complete the web-based questionnaire proved feasible. Participants reported that the length of the questionnaires was acceptable. The sending of emails was automated and easily administered and monitored via an online system. During app installation, a researcher was always present to help with any technical issues or answering questions. For the initial app installations, both technical and research team members attended to observe how participants perceived the installation process. Overall, the app installation worked as expected; however, based on observations in the pilot study, a few explanatory sections were added to the installation manual to be followed in the RCT. On average, the app installation and introduction to the wristband and selfBACK system took about 45 min.

## Discussion

The present pilot study confirmed that the recruitment procedure, the app installation procedure, and the data collection of outcomes via a web-questionnaire were feasible. The screening procedure was adjusted during the pilot as the PROMIS-PF4 questionnaire showed to be too restrictive, resulting in exclusion of potentially eligible participants. The intervention resulted in a moderate improvement in pain-related disability assessed by the RMDQ, which serves as the primary outcome in the subsequent RCT. Likewise, most secondary outcomes (e.g., pain intensity, fear-avoidance beliefs, and pain self-efficacy) improved but numerical changes were small. Time pattern of app usage varied considerable between the participants.

The suggested minimal clinically important difference in RMDQ score has been shown to vary according to the level of disability in the target population [21]. A 1–2-point reduction has been suggested to be clinically important in populations with low disability levels [45], while others have suggested that a relative reduction of 30% (regardless of level of disability) indicates a minimal clinically important difference [46]. Thus, the 1.8-point reduction in RMDQ score observed in this pilot study is relatively modest regardless of method for determining the minimal clinically important difference. However, it is important to recognize that this pilot study only assessed the within-group change from baseline to 6-week follow-up. The selfBACK RCT will investigate the effectiveness of the app in addition to usual care using the RMDQ as primary outcome measured at 3 months compared to a control group receiving usual care only.

The pilot study showed that the flow of participants referred to the study varied considerably both between countries, type of clinics and clinicians, and the clinicians response to being prompted to recruit rather than the clinicians professional background or the clinical setting. The average number needed to screen to include one participant was 1.6 but ranged from 1.3 to 1.9 between professions. In the RCT, the inclusion criterion for mild-to-severe disability will be assessed by the RMDQ using a score of ≥ 6 to indicate eligibility. Thus, we expect that the number needed to screen will increase slightly in the RCT. Furthermore, for the RCT, we will make some changes to the screening questions related to the other inclusion/exclusion criteria to increase participants understanding of the requirements for participating. Overall, the procedure for recruiting care-seeking patients with LBP from primary care was considered feasible for the RCT.

The pilot study also aimed to investigate the web-based data collection and app installation process. As a part of this process, members of the technical team attended some of the app installations on the different mobile devices brought by the pilot participants. Here, any questions from the participants concerning technical aspects such as understanding the log-in process, the functionality of the app, and participants’ intuitive responses to interpretation of app content, could be registered by the technical team or researchers and discussed with the participants. This allowed us to assess whether any revisions of the app installation procedure was necessary before carrying out the RCT. The comments did not result in any changes to functionality of the app. However, the explanatory text in the app given to participants during their initial log-in was revised, and additional explanation from the researchers was given to the users during installations. For example, text on how to report on exercise was revised and explanations concerning data handling and security and functionality of the step count and synchronization between wristband and app were elaborated on in the installation process. Overall, the pilot study led to two specific changes for the methods for the RCT; (1) the RMDQ questionnaire substituted the PROMIS-PF4 questionnaire as screening tool, and (2) the explanation of app functionality given to participants during app installations was modified.

## Conclusion

Pilot participants engaged with the app on a weekly basis and reported high achievement scores within all three content categories: physical activity, exercise, and education. The procedure for recruiting care-seeking patients with LBP from primary health care was feasible. Screening for eligibility on the phone was feasible but led to a change in choice of screening questionnaire from the PROMIS-PF4 questionnaire to the RMDQ. Collection of primary and secondary outcomes by a web-questionnaire proved feasible. The pilot study is followed by a multinational RCT with a two-armed design investigating the effectiveness of the app in a care-seeking LBP population.

## Supplementary information

**Additional file 1.** Supplementary Tables.

**Additional file 2.** Screening document.

## Data Availability

The datasets used and/or analyzed during the current study are available from the corresponding author on reasonable request.

## Abbreviations

BIPQ: Brief illness perception questionnaire
BMI: Body mass index
CBR: Case-based reasoning
CI: Confidence intervals
CONSORT: Consolidated Standards of Reporting Trials
DSS: Decision support system
FABQ: Fear avoidance-belief questionnaire
GPE: Global perceived effect
LBP: Low back pain
NRS: Numerical rating scale
PHQ-8: Patient health questionnaire 8-items
PSEQ: Pain self-efficacy questionnaire
PSFS: Patient Specific Function Scale
PSS: Perceived Stress Scale
QoL: Quality of life
RCT: Randomized controlled trial
RMDQ: Roland-Morris Disability Questionnaire
SD: Standard deviation
SGPALS: Saltin-Grimbye Physical Activity Level Scale
WAI: Work ability index
