# Supplementary material for: A digital decision support system (selfBACK) for improved self-management of low back pain: a pilot study with 6-week follow-up
Source: Pilot Feasibility Stud. 2020 May 23;6:72. doi: 10.1186/s40814-020-00604-2 (PMC7245029; doi:10.1186/s40814-020-00604-2)
Supplement: Supplementary file 1 — Additional file 1. Supplementary Tables. [file 40814_2020_604_MOESM1_ESM.docx]

**Table 1** Scores for self-reported outcomes at baseline and 6-weeks follow-up.

|  | Baseline (n=51)  Mean (SD) | 6 weeks (n=43)  Mean (SD) | Change score (n=43)  Mean (95% CI) |
| --- | --- | --- | --- |
| RMDQ | 8.6 (5.1) | 5.9 (4.0) | -1.8 (-2.9 to -0.7) |
| LBP intensity  Average past week  Worst past week | 4.1 (2.1)  5.7 (2.1) | 2.8 (1.8)  4.6 (2.5) | -1.0 (-1.6 to -0.5)  -1.0 (-1.6 to -0.4) |

| FABQ | 10.1 (5.6) | 8.3 (5.4) | -1.8 (-3.2 to -0.3) |
| --- | --- | --- | --- |
| FABQ LBP cause | 2.8 (2.0) | 2.0 (2.0) | -0.8 (-1.4 to -0.3) |

| PSEQ | 46.8 (11.1) | 50.6 (8.3) | 2.0 (0.4 to 3.6) |
| --- | --- | --- | --- |

| Work ability index* | 7.3 (2.2) | 7.4 (2.0) | -0.2 (-0.8 to 0.5) |
| --- | --- | --- | --- |
| PSFS | 3.7 (2.3) | 4.7 (2.7) | 1.0 (0.2 to 1.7) |
| EuroQol, 100mm VAS | 65.5 (14.9) | 75.0 (14.7) | 9.2 (4.4 to 13.9) |
| PHQ-8 | 6.3 (4.6) | 5.0 (3.7) | -0.9 (-2.1 to 0.4) |
| PSS | 14.0 (5.6) | 12.9 (6.6) | -0.9 (-2.6 to 0.8) |
| BIPQ | 47.7 (10.8) | 44.8 (10.8) | -2.7 (-5.4 to 0.0) |

Abbreviations: *SD* Standard Deviation; *CI* Confidence Interval; *RMDQ* Roland Morris Disability Questionnaire; *LBP* Low Back Pain; *FABQ* Fear Avoidance Belief Questionnaire; *PSFS* Patient Specific Function Scale; *PSEQ* Pain Self-Efficacy Questionnaire: *QoL* Quality of Life, *VAS* Visual Analogue Scale; *PHQ* Patient Health Questionnaire; *PSS* Perceived Stress Scale; *BIPQ* Brief Illness Perception Questionnaire.

*The question about work ability was only asked to participants who reported to be in full-time or part-time work

**Table 2** Number of pain sites at baseline and 6-weeks follow-up.

|  | Baseline (n=51)  Median (range) | 6 weeks (n=43)  Median (range) |
| --- | --- | --- |
| No. of pain sites | 3 (1 to 9) | 2 (0 to 6) |

Abbreviations: *LBP* Low Back Pain.

**Table 3** Scores for self-reported outcomes at baseline and 6-weeks follow-up.

|  | Baseline (n=51)  n (%) | 6-weeks (n=43)  n (%) |
| --- | --- | --- |
| Activity limitation  Not at work, not at leisure  Not at work, yes at leisure  Yes at work, not at leisure  Yes at work, yes at leisure | 5 (10)  11 (22)  1 (2)  34 (66) | 19 (44)  3 (7)  0 (0)  21 (49) |
| Physical Activity Level  Sedentary  Some physical activity  Regular physical activity  Regular hard physical activity | 1 (2)  27 (53)  20 (39)  3 (6) | 1 (2)  24 (56)  16 (37)  2 (5) |
| Sleep, feeling sleepy during the day  Seldom or never  Sometimes  Several times a week | 6 (12)  14 (27)  31 (61) | 6 (13)  17 (40)  20 (47) |
| Sleep, difficulties falling asleep at night  Seldom or never  Sometimes  Several times a week | 12 (24)  22 (43)  17 (33) | 14 (33)  17 (40)  14 (28) |
| Sleep, waking up repeatedly during night  Seldom or never  Sometimes  Several times a week | 14 (28)  21 (41)  16 (31) | 19 (44)  12 (18)  12 (18) |
| Sleep, waking up too early  Seldom or never  Sometimes  Several times a week | 6 (12)  17 (33)  28 (55) | 9 (21)  16 (37)  18 (42) |
| Patient Acceptable Symptom State  No  Yes | N/A  N/A | 23 (53)  20 (47) |
| Global Perceived Effect  Very much worse  Somewhat worse  Slightly worse  No change  Slightly better  Somewhat better  Very much better | N/A  N/A  N/A  N/A  N/A  N/A  N/A | 2 (5)  0 (0)  3 (7)  13 (30)  14 (33)  8 (19)  1 (2) |
