# Supplementary material for: A digital decision support system (selfBACK) for improved self-management of low back pain: a pilot study with 6-week follow-up
Source: Pilot Feasibility Stud. 2020 May 23;6:72. doi: 10.1186/s40814-020-00604-2 (PMC7245029; doi:10.1186/s40814-020-00604-2)
Supplement: Supplementary file 2 — Additional file 2. Screening document. [file 40814_2020_604_MOESM2_ESM.pdf]

OPEN SCREENING DOCUMENT

Note patient information in screening log

Oral information on project:

- Have you received the written patient information? (link in email text at baseline)
- Purpose of project: to investigate the effect of using an app to self-manage LBP
- Methods
    - Study design: randomisation to two groups, randomly assigned. Intervention period 9 mths
    - usual care group: seek and use care as normal
    - SELFBACK + usual care group: seek and use care as normal + use the selfBACK app to self-manage the LBP.
    - requirements: wearing wristband, downloading 3 apps, use mobile data in moderate amount (~20 MB pr. Week, if using both wifi and mobile data), data (including email address kept in Norway, but extensive precautionary measure are taken to ensure data security).
- Outcome assessment online, initial first visit at University.
- Rights: Volunteering to participate, withdraw at any time.
- Benefit of project: if the app is effective, it will be additive tool to primary care, and a tool readily available at the patient's convenience.

**Declaration of interests**	**Yes**	**No**
If you are interested in participating in the study, I'll ask you a series of questions to determine if you're able to be included.		

Screening questions

**Inclusion criteria's self-reported by participant**	**Yes**	**No**
What is your birth year?<br>If the year is 2000, please check if the participant if over 18 years of age.		
Do you have low back pain?		
Have you consulted a health-care professional within the past 8 weeks for your low back pain?		
Do you own and regularly use a smartphone?		
Is your operative system on your smartphone either Android 6.0 or iOS11.0+?<br>Which phone do you have? Which model do you have? How old is your phone? Can you check this in your phone settings? If not, then we need to check this on site before randomisation.		
Do you have access to internet on a daily basis via your smartphone – either via Wi-Fi or preferable a mobile data connection?		
Do you have a working email address and access to a computer with internet access?		

I'm going to ask you a series of questions about the level of difficulty you experience in different activities. For each question, you have 5 possible answers: Without any difficulty; With a little difficulty; With some difficulty; With much difficulty; or Unable to do

PROMIS-PF-4:	Without any difficulty	With a little difficulty	With some difficulty	With much difficulty	Unable to do
Are you able to do chores such as vacuuming or yard work?	5	4	3	2	1
Are you able to go up and down stairs at a normal pace?	5	4	3	2	1
Are you able to go for a walk of at least 15 minutes?	5	4	3	2	1
Are you able to run errands and shop?	5	4	3	2	1

*NOTE: complete all inclusion criteria, only if eligible proceed to exclusion. Ask all exclusion criteria questions

**Exclusion criteria's - self-reported by participant**		
Are you able to speak, read and write English?		
Do you have a cognitive impairment or a learning disability that may limit your participating in the study?		
Do you have an illness or condition such as a fracture, cancer, an inflammatory disease or signs of radiculopathy?*		
*(Severe leg pain, loss of leg strength, or loss of or altered sensation in a myotomal or dermatomal distribution)		
Do you have a serious mental illness that may limit your participating in the study?		
such as major depression, schizophrenia, and psychosis		
Do you have a terminal illness?		
Are you able to take part in exercise and physical activity?		
For example, are you able you able to walk without using walking aids or assistance? Are you able to get up and down from the floor independently?		
Have you been diagnosed by a doctor as having fibromyalgia?		
Are you currently pregnant?		
Have you ever had back surgery?		
Are you enrolled in other research trials for low back pain management?		

**Verbal consent**	**Yes**	**No**
You have fulfilled all the criteria for being included in the study. Are you interested in taking part in the study?		

Set-up visit with patient
Note in excel document

Preparing for enrolment

- Generate patient id no in SELFBACK system
- Send email with link to baseline questionnaire to patient
- **Update screening information**
